# Developments and Clinical Applications of Noninvasive Optical Technologies for Skin Cancer Diagnosis

**DOI:** 10.1155/2022/9218847

**Published:** 2022-11-18

**Authors:** Hamza Abu Owida

**Affiliations:** Medical Engineering Department, Faculty of Engineering, Al Ahliyya Amman University, Amman 19328, Jordan

## Abstract

Skin cancer has shown a sharp increase in prevalence over the past few decades and currently accounts for one-third of all cancers diagnosed. The most lethal form of skin cancer is melanoma, which develops in 4% of individuals. The rising prevalence and increased number of fatalities of skin cancer put a significant burden on healthcare resources and the economy. However, early detection and treatment greatly improve survival rates for patients with skin cancer. Since the rising rates of both the incidence and mortality have been particularly noticeable with melanoma, significant resources have been allocated to research aimed at earlier diagnosis and a deeper knowledge of the disease. Dermoscopy, reflectance confocal microscopy, optical coherence tomography, multiphoton-excited fluorescence imaging, and dermatofluorescence are only a few of the optical modalities reviewed here that have been employed to enhance noninvasive diagnosis of skin cancer in recent years. This review article discusses the methodology behind newly emerging noninvasive optical diagnostic technologies, their clinical applications, and advantages and disadvantages of these techniques, as well as the potential for their further advancement in the future.

## 1. Introduction

Skin cancer has shown a sharp increase in prevalence over the past few decades and currently accounts for one-third of all cancers diagnosed [1]. There is a 4% chance that a person will acquire melanoma, the deadliest form of skin cancer, due to its propensity for metastatic spread and invasion [2]. The rising prevalence and mortality rates of skin cancer put a significant burden on healthcare resources and the economy. However, early detection and treatment greatly improve survival rates for people with skin cancer. Basal cell carcinoma (BCC), the most frequent type of nonmelanoma skin cancer, and squamous cell carcinoma (SCC), the second most common type, make up the nonmelanoma category. Cancers of this type typically manifest in the face, ears, neck, and arms due to their exposure to the sun but can develop anywhere on the body. Although it is uncommon for BCCs to spread to other parts of the body, they can do so if left untreated because of their slow growth rate. SCCs are more aggressive than other types of skin cancer and can invade deeper skin layers and metastasize to other parts of the body [3–6].

Visual inspection, followed by a specimen and histopathologic evaluation, is the standard procedure for diagnosing melanoma. The “ABCDE rule” is the standard by which visual inspection in this field is judged, which describes the symptoms of the most frequent forms of melanoma: its asymmetry (*A*), border irregularity (*B*), color variation (*C*), dimension higher than 6 mm (*D*), and evolution (*E*) are all characteristics that may be described using the letters *A*, *B*, *C*, *D*, and *E* [7]. However, due to the fact that it is an opinionated evaluation of the injury that is greatly reliant on the expertise of specialists, roughly one-third of MM would be missed merely by looking at it with the naked eye. This is because it is extremely dependent on the experience of physicians. In particular, melanomas in their early stages are more likely to go undetected due to the fact that the tumors are typically rather small and lack other distinguishing characteristics [6, 7].

Since histological evaluation is the gold standard, it necessitates surgical excision of the tumor (biopsy), adding to substantial direct annual costs associated with treating skin cancer [8]. Expert surgeons are required to administer anesthetics, create the incision, and oversee the entire operation. The sample is then examined under a microscope by a histology expert who will treat it and determine what is wrong. Not only is it expensive, but the process can take up to three weeks from the time a lesion is removed until a definitive diagnosis is reached [9] because of the involvement of several seasoned physicians.

**Figure 1:**
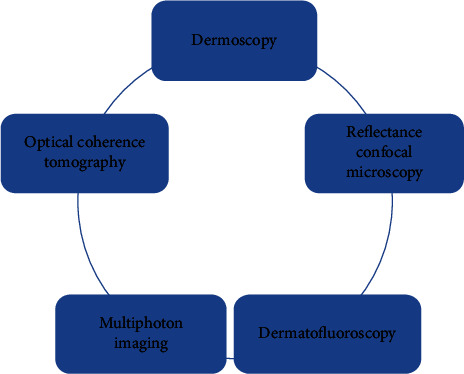
Noninvasive optical technologies for skin cancer diagnosis.

Even though histopathology is the gold standard for diagnosing skin cancer, people often refuse to undergo the procedure because of its invasive nature. Therefore, improving diagnostic accuracy and reducing the number of needless biopsies are of clinical importance. Technology is being developed further to aid in the search for a more objective and noninvasive optical form of diagnosis, and great strides have been made in this direction [10] (Figure 1).

This study is meant to provide a comprehensive assessment of numerous noninvasive optical technologies for diagnosing skin cancer, including a discussion of the advantages and disadvantages of these technologies, their clinical application, and the potential for further development of these technologies in the future.

## 2. Dermoscopy

In terms of noninvasive imaging techniques, dermoscopy has been around for quite a long period. The term “dermoscopy” was used in the 1950s to describe a new method for assessing pigmented skin lesions (PSLs). It bridges the gap between clinical and histopathologic evaluation [11] by the provision of horizontal images of subsurface structures at magnifications ranging from 10 to 20 times at various layers. As a result, it is now considered a primary diagnostic method. In response to the growing need for image data storage and access, digital dermoscopy was created. As a result of these advancements, new types of image analysis software have been developed to aid in the interpretation of these images. They enable risky patient evaluation and diagnosis with computer assistance or without human intervention, two of dermoscopy's key potential applications, which would bolster the significance of dermoscopy [11].

Its clinical application was first seen in 1987, and since then, it has become the most popular. Despite its widespread acceptance and reputation for dependability, its diagnostic accuracy continues to rely on the judgment of trained professionals rather than medical students [13]. Nowadays, various key advancements, methods have been developed to streamline diagnosis, guidelines of dermoscopy [14], Menzies method [15], 7 point score [16]. Such enhancements have been widely used since their 2001 endorsement at the CNMD [17].

Observed meta-analyses have shown that dermoscopy is an effective adjunct in MM detection. Evidence suggests that dermoscopy improves MM diagnosis accuracy by 49% relative to an unassisted visual assessment and that seasoned clinicians can be educated to reach a diagnostic accuracy by about 80% with dermoscopy [18–20]. This has led to its widespread clinical use. Dermoscopy is useful in the diagnosis of MM because it helps reduce the number of unnecessary biopsies performed. Dermoscopy has been shown, through a 10-year, multicenter study (1998–2007), to reduce surgical morbidity by preventing the biopsy of benign lesions [21]. In order to detect MM as early as possible, it is best to use “two-stepfollow-up techniques,” which automated dermoscopy and entire skin scanning following development of malignant tumors.

Dermoscopy helps diagnose MM. Missing or unclear morphological patterns might cause misinterpretation. More activities are concentrated on establishing optimum procedures or criteria for subgroups of MM that are difficult to diagnose [23]. To date, dermoscopy has achieved widespread clinical approval and can be compared to the stethoscope for dermatologists. However, due to its subpar detection depth and resolution, it is still no match for histology. In addition, dermoscopy cannot be performed on vertical tissue sections. To overcome these restrictions, alternative optical approaches could be utilized in tandem with dermoscopy.

It is important to note that dermoscopy is still very much a work in progress. The main focus of modern dermoscopy studies is on teledermoscopy and automatic diagnosis software. Teledermoscopy is a subset of teledermatology that expedites patient referral. Patients can avoid the expense and inconvenience of traveling to specialized clinics by using this method. Seeing as there is no difference between in person and remote diagnosis when carried out by professionals [24, 25].

Another option for assessment and/or monitoring of lesions is patient-performed mobile teledermoscopy [26, 27] that has recently been investigated, thanks to the introduction of mobile applications and cheap attachments for smartphones.

The medical industry is currently undergoing significant change and influence from artificial intelligence. First demonstrated in 1994 by Binder et al. [28], AI can identify MM from dermoscopy data solely. The advancement of numerous picture databases in addition to open source software has allowed for significant improvement in automated dermoscopy. The largest publicly available collection of dermoscopy images has marked a significant step forward for the use of machine learning models for the diagnosis of MM [29]. More recently, it has been shown that AI is superior to human experts, suggesting that AI can play a vital role in clinical practice [30].

The available algorithms also have only a moderate degree of diagnostic accuracy, and there is a steep learning curve to get up to speed. It is currently widely believed that artificial intelligence is the primary answer to the first issue. The growing availability of data and improvements in diagnostic tools have allowed even laypeople to improve their diagnostic prowess. When designing improved analytical algorithms, it may be possible to factor in additional information, such as the lesion's evolutionary changes and the patient's medical background. In addition, advancements in both hardware and software for dermoscopy are required to enhance the quality of care provided to patients. In addition to decreasing in size and increasing in usability, these tools need to have more sophisticated features, such as the ability to upload photographs to a patient's medical record in real time. More work needs to be performed to improve the integration of teledermoscopy with cell phones, such as the creation of user friendly software that allows patients to easily and consistently take and upload high-quality photos.

## 3. Multiphoton Microscopy

With subcellular resolution, multiphoton microscopy can examine the skin to a depth of 200 micrometers. Aspects of fluorescence activated by multiple incident light signals can be used to detect endogenous fluorophores such as nicotinamide adenine dinucleotide (NADH) and flavin adenine dinucleotide (FAD). Because of this, multiphoton imaging can reveal an unstained lesion's function and structure [31]. It is possible to learn something from fluorescence by analysing both its intensity and duration. Therefore, fluorescence lifetime imaging and multiphoton imaging can be used interchangeably (FLIM). Potentially useful for early diagnosis of malignant lesions [32], changed FLIM signals of malignancies can be attributable to numerous causes. Multiphoton imaging has various benefits over conventional linear optical techniques. Nonlinear signals require an abundant supply of photons, making them extremely location dependent, which means you can do optical sectioning without resorting to a confocal pinhole. Longer wavelengths mean greater depth of penetration. It can reveal details on the lesion's microenvironment and fluorophore activity.

When comparing the fluorescence properties of MM and nevi, clear morphological distinctions were discovered. Six diagnostic markers of MM include escalating melanocytes, a large interstitial gap, structural instability, imprecise keratinocyte cell borders, cell eosinophilic cytoplasm, and progenitor cells [33]. The human skin's first MPT, DermaInspect® (JenLab, Jena, Germany), was developed in 2002. It has the ability to instantly capture MPT and FLIM signals in vivo. In 2009, Dimitrow et al. evaluated DermaInspect in a clinical investigation, finding that it had a sensitivity of 71%–95% and a specificity of 69%–97% [33].

In ex vivo samples, Seidenari et al. investigated how well MPT combined with FLIM performed. The diagnostic sensitivity and specificity for MM were both 100%. High-quality evidence, however, cannot be collected without first conducting massive clinical studies [32].

In addition to its many advantages, multiphoton imaging does have some major downsides. Due to its weak signals, multiphoton imaging requires stronger lasers and longer detection periods. Motion artifacts are plainly visible in its high-resolution photographs. Its utility is constrained by its high price. For the time being [34] to get over these limitations, accumulating research indicates that the combination is useful in identifying scars, nevi, and BCC.

## 4. Reflectance Confocal Microscopy

It is possible to examine a lesion in real time with cellular precision using reflectance confocal microscopy (RCM) down to a penetration that is about equivalent to the upper papillary dermis (200 m). This technology works by using a pinhole and a filter to prevent reflection from the outside of the focus area and selectively excite a specific point with near-infrared (NIR) light. Taking a series of photographs at progressively deeper levels parallel to the skin's surface, it provides a three-dimensional view of the lesion [35]. By comparing the reflection indices of various skin components, RCM pictures can reveal a lot of information about the skin's structure.

Nuclei and collagen are darker because they have low reflection indices [36], while melanin and keratin have high indices. Results from RCM are in lockstep with those from traditional histology [37].

RCM is very helpful in the diagnosis of MM because of the high reflection indices of melanin, and there are various distinguishing markers between MM and nevi [38–41]. Among the most popular methods are the scoring system by Pellacani et al. [39] and the two-stage process by Segura et al. [40]. Most dermal MMs can be diagnosed with their assistance. However, they should not be used for in situ melanoma at this time (MIS), which are epidermally localized MMs. There is also a two-stage scorecard, created by Bosari et al. [41], which fills in this void. These grading systems can aid in the implementation of RCM in MM diagnosis and are of particular use to inexperienced clinicians.

Commonly utilized is the VivaScope® 1500 (Caliber Imaging and Diagnostics, Inc., NY, USA.), a dermoscope equipped, wide probe RCM instrument [42]. It can take photos with a resolution of 0.5 mm × 0.5 mm, which can then be combined to create an 8 mm by 8 mm image.

However, because it must adhere to the skin in order to function, the device has problems detecting slight or irregular areas and is influenced by skin condition [42]. Fortunately, a number of adaptations of devices are made for their use in a variety of medical settings. For instance, smaller curved surfaces, like the face, can be detected with the help of handheld equipment like VivaScope® 3000 [42].

Clinically and dermoscopically ambiguous lesions can benefit from RCM's usage as a secondary diagnostic tool because it helps prevent unnecessary biopsies [39]. Indications for RCM were further elucidated by Borsari et al. [43], who specified head and neck lesions, regressing lesions, and chronically sun-damaged skin as appropriate sites. Besides identifying MM, RCM can be used to assess recurrence, monitor noninvasive skin therapies, and make sure that clean margins are defined before surgery [44–46].

There are several clinical uses of RCM, making it a viable supplementary tool. Although RCM has been around for a while and is recognised, few specialists apply it to their patients regularly [47], which is possibly due to following technical restrictions. First, its usefulness for widespread and/or deeply embedded lesions is restricted by its shallow penetration and narrow detection field. Second, when there is an abundance of highly reflective contents (such as ulceration, hyperkeratosis, or even supplemental treatments like sunscreen) in the superficial layer, image resolution and detection depth get drastically reduced. Finally, a great deal of clinical expertise and experience is needed for accurate interpretation of RCM pictures. For RCM to be widely used in clinical settings, especially for primary care, advancements in portable, lightweight, and accurate instruments are needed.

Researchers are actively looking for new avenues of progress right now. Similar to dermoscopy, software-computerized detection of RCM is now in progress and holds great potential [48–50]. These kinds of computational models could one day yield quantitative tools for guiding standardized transcription of obtained images and for providing platforms for training and teaching. In addition, advancements could be made in fluorescence confocal microscopy (FCM). Confocal microscopy with fluorescent agents is used to improve skin contrast [44, 51].

FCM is largely employed in in vivo specimens for perioperative operative perimeter evaluation, and its use is exploratory. Researchers are studying its application in MM [52].

## 5. Dermatofluoroscopy

The presence of melanin is what makes MM unique. Sadly, its feeble signal is easily disrupted by other fluorophores, making it nearly undetectable using conventional one-photon-stimulated fluorescence [53]. On top of that, there are no clear peaks in the melanin spectrum that may be used for analysis [54]. The use of a nanosecond-pulsed laser, as opposed to a femtosecond-pulsed laser, has allowed for the development of step-by-step two-photon ingestion, an unconventional form of emission, which can circumvent these drawbacks [55, 56]. This allows for selective excitation of melanin, leading to a fluorescence spectrum, which is dominated by melanin emission. The term “dermatofluoroscopy” was coined to describe the technique. The fluorescence in MM has a noticeable red shift in the fluorescence peak [55, 56].

Magnosco DermaFC®, developed by Magnosco GmbH in Berlin, Germany, allows for in vivo diagnosis of PSLs. Pulses from the gadget can penetrate the skin up to 500 micrometers and illuminate an area of 50 micrometers in diameter with a wavelength of 810 nanometers. Its scanning head can measure spots one by one across an area of up to 20 by 20 mm, and it can acquire a series of melanin fluorescence spectra that reflect the extent of malignancy. After all of these spectra have been sorted and examined with an in-built, objective, and automated data processing system, a score is then produced to help distinguish MMs from other PSLs [57]. In a prospective, blinded, multicentre study that detected 476 PSLs, with a cutoff score of ≥30, sensitivity and specificity were 89.1% and 44.8%, respectively [58].

Because it does not rely on the individual characteristics of the patient, dermatofluoroscopy is more accurate and precise than other noninvasive procedures. Although dermatofluoroscopy offers many benefits, it also has some major drawbacks. Light colored or rapidly regressing melanocytic lesions, such as AHM, are not good candidates for this treatment. It also lacks the ability to reveal details regarding the lesion's thickness. Therefore, it has severe constraints.

## 6. Optical Coherence Tomography

With optical coherence tomography (OCT), an interferometric imaging technique is used to create three-dimensional images. The lesion's structure and alterations within the skin are studied using an infrared broadband light source. By using an interferometer, the beam of light is separated into a sample arm and a reference arm. In order to recombine the sample signal with the reference signal, the sample arm is directed at the lesion's target region and then reflected back. Both beams' pathlengths must be within the light's coherence length for interference to take place [59].

The interference signal is analyzed by OCT, allowing for the acquisition of cross-sectional pictures in real time, with a resolution of 315 m and a depth of 1.52 mm [59]. Optical coherence tomography (OCT) performs better than other methods because it can reach the lesion's deep boundaries and display horizontal images simultaneously by fusing all cross-sectional images into one. Therefore, since its initial introduction in 1995, OCT has seen steadily expanding applications in dermatology.

Although conventional OCT has showed promise in the detection of nonmelanoma skin malignancies [60], it is widely held that its relatively low resolution and poor picture quality make it unsuitable for MM diagnosis. As a result, more advanced OCT systems including high-definition OCT (HD-OCT) and dynamic OCT (D-OCT) have been created to circumvent these constraints. Although research on them is still in its infancy, they hold promise as a means of distinguishing PSLs and providing additional features.

Images with a cellular resolution of 3 *μ*m are possible with HD-OCT such as Skintell® (Agfa Healthcare, Mortsel, Belgium) [61], but this comes at a cost of a shallower penetration depth of 750 m and a smaller scan area of 1.5 1.5 mm^2^ than what is ideal. HD-OCT traits have been shown to significantly correlate with RCM or histopathological findings. The invasion of atypical melanocytes gives MMs a more disorganized appearance than benign nevi [62]. HD-OCT offers a wider detection field and greater penetration depth than RCM, allowing it to obtain more detailed information than standard OCT. Multiple research studies found that although HD-OCT's specificity was quite high at 92.4%, its sensitivity was relatively moderate at 74.1%. The high false-negative rate in thin melanomas and the high false-positive rate in dysplastic nevi [63] may be because the diagnosis by HD-OCT mostly relies on examining the tumor's thickness and the borderline of the lesion. Rather than relying solely on morphological analysis, researchers are now seeking to boost precision by examining optical features in greater depth [64].

D-OCT is a speckle-variance OCT (SVOCT)-based functional OCT that allows for in vivo observation of skin microvasculature. The technology relies on OCT scans that are repeated at high speeds and analyzed in real time. The diagnostic utility of D-OCT can be enhanced by gaining insight into the vasculature of skin illnesses due to the modest changes in data that blood flow causes to disclose their presence [65]. Vascularity is seen on D-OCT in pigmented nevi as discrete, regularly spaced dots. However, in MM, these vessels typically show a denser and chaotic distribution, with irregular cylindrical shapes in the vertical section [66]. It has been found that D-OCT can aid in the prognosis of MM patients. It has been shown that microvascularization is highly correlated with the Breslow index. A higher score is associated with a less regular vascularization pattern on D-OCT [67]. Currently, D-OCT may be performed with a 6 mm × 6 mm field of vision using Vivosight® (Michelson Diagnostics, Kent, United Kingdom). With an axial resolution of 7.5 m and a lateral resolution of 5 m, it is able to provide a clear presentation of the extracellular matrix and microcirculation [68].

However, OCT's poor resolution and the optical characteristics of melanin have prevented it from becoming useful in MM diagnosis. D-OCT and H-DOCT are promising emerging technologies that may increase diagnostic precision by supplementing OCT with additional data. However, these methods are in in their infancy, and further research is needed to confirm their usefulness in MM diagnosis. There is a clear need for future improvement in their obvious utility, and thus, the development of associated automated identification and classification software is likewise warranted.

## 7. Conclusion

Skin lesions, in contrast to conditions affecting other systems, lend themselves particularly well to the application of noninvasive procedures, therefore encouraging the development of new technologies. These optical approaches can greatly improve the inspectional part of traditional diagnosis, but they cannot replace the gold standard, which is a biopsy followed by histopathologic examination. They have been shown to be helpful in achieving optimal MM management. These examinations are generally more sensitive, objective, and socially acceptable than the traditional eye exam, even among nonspecialists. An optical characteristic analysis of tissue can also give information about the tissue's internal architecture, biological components, and metabolic changes, which are otherwise hidden to the naked eye. These developments may help reduce the frequency of unnecessary excisions and enable dependable long-termfollow-ups for high-risk patients. A second use with the potential to greatly enhance the prognosis of patients with MM is non invasive presurgical evaluation.

The two key instruments that have previously been used extensively in a wide variety of clinical settings across the globe today are dermoscopy and RCM. Both multiphoton imaging and stepwise two-photon fluorescence have commercially available solutions; however, there is still need for improvement in both areas before they can be employed more broadly in either primary care or specialty care settings. OCT has a lot of space for development despite having two promising derivative techniques at its disposal.

Optical methods have showed great promise for MM diagnosis, but there is still much need for improvement and exploration. Improvements in diagnostic precision, detection speed, portability, and device cost effectiveness are all required. In the not-too-distant future, more advanced technologies may be developed to facilitate efficient data collection. Portable technologies and approaches based on cellphones may make remote diagnostics feasible in the future. Dermatologists may find computer-aided diagnosis or AI-based technology to assess optical data and make automated, objective diagnoses helpful in screening lesions before referral.  Table 1 presents advantages, disadvantages, and commercially available technologies for different noninvasive optical methods.

## Figures and Tables

**Table 1:** Summary of optical techniques for skin cancer diagnosis.

Diagnostic technique	Commercially available technologies	Advantages	Disadvantages
Dermoscopy	Delta® 20 T Dermlite® Veos®	Better overall diagnostics, prior surgical intervention	Low quality, little depth of engagement, extra instruction needed
Multiphoton microscopy	DermaInspect®	Confinement of data to a single layer, extremely fine resolution down to the subcellular level, wide area coverage	Low signal strength, high acquisition time, susceptibility to artifacts introduced by motion, high cost
Dermatofluoroscopy	DermaFC®	Spectra of melanin are extremely sensitive, allowing for a scientifically sound detection process	Lesion depth information is lacking; only pigmented lesions can be detected
Reflectance confocal microscopy	Vivascope	Extensive correlation with histology, high precision, cellular resolution, better contrast image	Insufficient detecting range and depth, as well as a lengthy learning curve, important to have healthy skin, expensive, needs lots of practice to be effective
Optical coherence tomography	Vivosight® Skintell®	Intense imaging, fast picture capture, vertical imaging; imaging of vasculature	Inadequate resolution (with the exception of HD-OCT), expensive, preferable used on healthy skin, extra instruction needed

## Data Availability

The data that support the findings of this study are available on request from the author.
